# Application of neck anastomotic muscle flap embedded in 3-incision radical resection of oesophageal carcinoma: A protocol for systematic review and meta analysis

**DOI:** 10.1097/MD.0000000000022263

**Published:** 2020-10-09

**Authors:** Zhangwei Tong, Xiaojie Yang, Fei Luo, Jiafu Zhu, Mingqiang Kang, Jiangbo Lin

**Affiliations:** Department of Thoracic Surgery, Fujian Medical University Union Hospital, Fuzhou, China.

**Keywords:** 3-incision, esophageal stomal leak, muscle flap of embedding, oesophageal cancer, radical resection

## Abstract

**Background:**

Oesophageal cancer is one of the most common malignant tumors and has been identified as one of the leading causes of cancer death worldwide. Surgery is considered to be the optimal treatment for patients with resectable oesophageal cancer. Oesophagectomy for oesophageal cancer can significantly extend the survival period of patients and provide a potential opportunity for a cure. However, there is still controversy regarding application of neck anastomotic muscle flap embedded. This systematic review and meta-analysis will be performed to determine whether the application of neck anastomotic muscle flap embedded would benefit patients more.

**Methods:**

We will search PubMed, Web of Science, Embase, Cancerlit, the Cochrane Central Register of Controlled Trials, and Google Scholar databases for relevant clinical trials published in any language before October 1, 2020. Randomized controlled trials (RCTs), quasi-RCTs, propensity score-matched comparative studies, and prospective cohort studies of interest, published or unpublished, that meet the inclusion criteria will be included. Subgroup analysis of the type of operation, tumor pathological stage, and ethnicity will be performed. INPLASY registration number: INPLASY202080059.

**Results:**

The results of this study will be published in a peer-reviewed journal.

**Conclusion:**

As far as we know, this study will be the first meta-analysis to compare the efficacy of the application of neck anastomotic muscle flap embedded in 3-incision radical resection of oesophageal carcinoma. Due to the nature of the disease and intervention methods, RCTs may be inadequate, and we will carefully consider inclusion in high-quality, non-RCTs, but this may result in high heterogeneity and affect the reliability of the results.

## 1 Introduction

Oesophageal cancer is one of the most common malignant tumors in the world, and its incidence rate ranks 7th among all malignant tumors.^[1]^ Oesophageal cancer has been identified as one of the leading causes of cancer death because of the high degree of malignancy and low survival rate of patients.^[2–4]^ Surgery is regarded as the best option for patients with resectable oesophageal cancer. Oesophagectomy for patients with oesophageal cancer can significantly extend the survival period and provide a potential opportunity for a cure.^[5–7]^

In the last dozen years, video-assisted thoracoscopic oesophagectomy has developed rapidly, and a variety of available technical approaches have been formed.^[8,9]^ Many trials have reported that video-assisted thoracoscopic oesophagectomy can bring more benefits to patients than traditional open thoracic oesophagectomy.^[10–13]^ However, there is still controversy regarding application of neck anastomotic muscle flap embedded. This systematic review and meta-analysis will be performed to determine whether the application of neck anastomotic muscle flap embedded would benefit patients more and to provide a basis for clinicians to develop optimal treatment strategies for patients.

### 1.1 Objective

We will conduct a systematic review and meta-analysis to estimate the efficacy and safety of the application of neck anastomotic muscle flap embedded in 3-incision radical resection of oesophageal carcinoma.

## 2 Methods

This protocol adheres to the preferred reporting items for systematic review and meta-analysis protocols statement.^[14]^ The results of this systematic review and meta-analysis will be published with reference to the preferred reporting items for systematic review and meta-analysis guidelines.^[14]^

## 3 Patient and public involvement

This study will be based on published or unpublished studies, and records and will not involve patients or the public directly.

### 3.1 Eligibility criteria

#### 3.1.1 Types of studies

Randomized controlled trials (RCTs), quasi-RCTs, propensity score matched comparative studies, and prospective cohort studies of interest, published or unpublished, will be included. These should be completed, and the efficacy and safety of the application of neck anastomotic muscle flap embedded or not in 3-incision radical resection of oesophageal carcinoma for patients with oesophageal cancer will be compared.

#### 3.1.2 Types of participants

The participants will be patients diagnosed with resectable, pathologically confirmed oesophageal cancer who were treated with video-assisted thoracoscopic oesophagectomy, and there will be no restrictions on sex, ethnicity, economic status, or education.

#### 3.1.3 Types of interventions

All types of video-assisted thoracoscopic oesophagectomy with application of neck anastomotic muscle flap embedded or not for patients diagnosed with resectable oesophageal cancer will be studied.

#### 3.1.4 Types of outcome measures

##### 3.1.4.1 Primary outcomes

The primary outcome will be overall survival of patients with resectable oesophageal cancer after surgery.

##### 3.1.4.2 Secondary outcomes

We will evaluate the 5-year survival, esophageal stomal leak, recurrence-free survival, and median survival rates as well as the quality of life and complication rate of patients with resectable oesophageal cancer after surgery.

### 3.2 Information sources

Two reviewers (ZWT and XJY) will search PubMed, Web of Science, Cancerlit, Embase, Cochrane Central Register of Controlled Trials, and Google Scholar databases for relevant trials published before October 1, 2020, without any language restrictions.

### 3.3 Search strategy

The subject terms and keywords corresponding to Medical Subject Heading terms will be used to search for eligible trials in the databases as mentioned above with no language restrictions. Search strategies in PubMed are shown in Table 1.

**Table 1:**
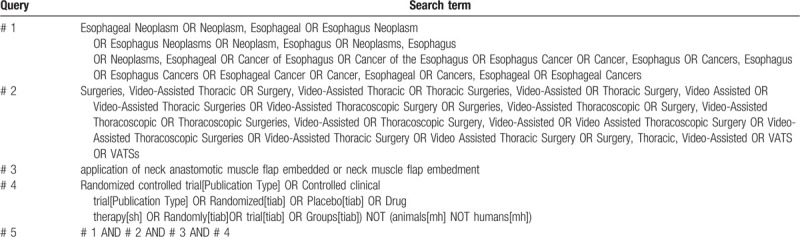
Pubmed search strategies.

### 3.4 Data collection and analysis

We will adopt the methods described in the Cochrane Handbook for Systematic Reviews of Interventions to pool the evidence.^[15]^

#### 3.4.1 Study selection

Two authors (ZWT and XJY) will independently screen each title and abstract of all the papers searched, and the trials that do not meet the inclusion criteria described in this protocol will be excluded. The full text of all the possibly eligible trials will be screened independently and in duplicate by the 2 authors. Trials that are irrelevant or do not meet the inclusion criteria will be excluded. Trials that meet the inclusion criteria and excluded studies along with the reasons for their exclusion will be documented by the 2 authors (ZWT and XJY). If there is a disagreement between the 2 authors, we will come to a resolution by discussing it with the third author (FL). If necessary, we will consult the fourth author (JBL) to resolve the disagreement. The selection process will be shown in a preferred reporting items for systematic review and meta-analysis flow chart in detail.

#### 3.4.2 Data extraction and management

We will extract the following data from the included trials.

- Study characteristics: author, publication date, country, study design, randomization, periods of data collection, follow-up duration, withdrawals, and overall duration of study.
- Population characteristics: age, sex, pathology diagnosis, tumor stage, pathologic tumor size, performance status, ethnicity, history of smoking, and inclusion criteria.
- Interventions: type of operation, number of lymph nodes retrieved, extent of resection, duration of operation, bleeding, and postoperative adjuvant therapy.
- Outcomes: overall survival, 5-year survival, recurrence-free survival, median survival, length of stay, length of intensive care unit stay, quality of life, complications, and adverse events.

We will use the pre-designed table to record the data extracted from the included trials. If relevant data from the trials are lost or unclear, we will consult the author via email before determining whether the study is to be included.

### 3.5 Assessment of risk of bias

The Cochrane Handbook for Systematic Reviews of Interventions will be used to assess the risk of bias of each trial included. The 2 authors (ZWT and XJY) will evaluate the risk of bias based on the following domains: random sequence generation (selection bias), allocation concealment (selection bias), blinding of participants and personnel (performance bias), blinding of outcome assessment (detection bias), incomplete outcome data (attrition bias), selective outcome reporting (reporting bias), and other biases.^[16]^ The risk of bias in each domain will be assessed as high, low, or uncertain, and the results of the evaluation will be shown on the risk of bias graph. EPOC guidelines will be used to assess the risks of the non-RCTs included.^[17]^

### 3.6 Data analysis

We will use Review Manager and Stata software to synthesize the data extracted. If the data extracted from the included studies are evaluated as highly homogeneous, we will use them to conduct a meta-analysis for the purpose of obtaining a clinically meaningful result. To carry out a standard meta-analysis, we will use the Chi^2^ and *I*^2^ statistical tests to evaluate statistical heterogeneity among the studies. If there is high heterogeneity (*P* < .1 or *I*^2^ statistic > 50%), we will use the DerSimonian and Laird random effect model to analyze the extracted data. Because high heterogeneity may be caused by different types of tumors and different stages of tumors diagnosed by pathology and different means of adjuvant therapy may be used after the operation, we will perform a subgroup analysis of the types of tumors (oesophageal squamous cell carcinoma and oesophageal adenocarcinoma), the pathological stages of the tumors, and the means of adjuvant therapy after the operation (types of chemotherapeutic drugs and whether or not radiotherapy is accepted). Otherwise, we will adopt a fixed-effect model to analyze the data. We will adopt the Mantel–Haenszel method to pool the binary data, and the results will be reported in the form of relative risk with a 95% confidence interval. An inverse variance analysis method will be used to pool the continuous data, and the results will be reported in the form of a standardized mean difference with a 95% confidence interval.

#### 3.6.1 Subgroup analysis

If there is substantial heterogeneity and if the available data are sufficient, we will perform subgroup analysis to search for potential origins of heterogeneity. If the extracted data are enough, we will conduct subgroup analysis of the type of operation, type of tumor, tumor stage, age, and postoperative adjuvant treatment.

#### 3.6.2 Sensitivity analysis

We will conduct a sensitivity analysis to evaluate the robustness and reliability of the aggregation results by eliminating trials with a high bias risk. If a reporting bias exists, we will use the methods of fill and trim to analyze for publication bias.^[18]^

### 3.7 Publication bias

Funnel charts and Egger test will be adopted to assess for publication bias if there are no less than 10 eligible trials. If reporting bias is suspected in a trial, we will contact the corresponding author via email to determine whether there are additional outcome data that were not reported.

### 3.8 Evidence evaluation

We will classify the quality of all the evidence into 4 levels (high, medium, low, and very low) in accordance with the criteria of grading of recommendations, assessment, development, and evaluation (study limitations, imprecision, publication bias, indirectness bias, and effect consistency).^[19]^

## 4 Discussion

Oesophageal cancer is one of the worst malignant digestive neoplasms and has poor treatment outcomes. Oesophagectomy is a major part of the treatment strategy for locally resectable oesophageal cancer and plays an important role in the treatment of patients with oesophageal cancer, providing a potentially curable opportunity for these patients.^[5–7,20]^ Video-assisted thoracoscopic oesophagectomy for patients with oesophageal cancer has been used worldwide, but controversy about the application of neck anastomotic muscle flap embedded in 3-incision radical resection of oesophageal carcinoma and esophageal stomal leak persists.

As far as we know, this study will be the first systematic review and meta-analysis to compare the efficacy and outcome of application of neck anastomotic muscle flap embedded in 3-incision radical resection of oesophageal carcinoma to provide a basis for clinicians to develop optimal treatment strategies for patients.

## Author contributions

**Conceptualization:** Zhangwei Tong, Xiaojie Yang, Fei Luo.

**Data curation:** Zhangwei Tong, Xiaojie Yang, Fei Luo.

**Formal analysis:** Zhangwei Tong, Xiaojie Yang, Fei Luo, Mingqiang Kang.

**Funding acquisition:** Mingqiang Kang, Jiangbo Lin.

**Investigation:** Zhangwei Tong, Jiangbo Lin.

**Methodology:** Zhangwei Tong.

**Project administration:** Zhangwei Tong.

**Resources:** Zhangwei Tong, Jiafu Zhu.

**Software:** Zhangwei Tong.

**Supervision:** Zhangwei Tong.

**Validation:** Zhangwei Tong.

**Visualization:** Zhangwei Tong.

**Writing – original draft:** Zhangwei Tong.

**Writing – review and editing:** Zhangwei Tong.

## Abbreviations

Abbreviation: RCTs = randomized controlled trials.
